# Association Between Environmental Risk Factors and Asthma Control Among Hospitalized Children With Recurrent Wheezing: A Cross-Sectional Study

**DOI:** 10.7759/cureus.88453

**Published:** 2025-07-21

**Authors:** Attique Manzoor, Hamza A Chaudhry, Jain Ambo, Shaheera Raghib, Saadia Karim, Sushmitha Reddy, Maria Arshad, Mahek Thorani, Muhammad Rahim Arshad, Fathima Farzana

**Affiliations:** 1 Medicine and Surgery, Faisalabad Medical University, Faisalabad, PAK; 2 Medicine, Combined Military Hospital (CMH) Lahore Medical College and Institute of Dentistry, Islamabad, PAK; 3 Pediatric Medicine, Yangtze University, Wuhan, CHN; 4 Pediatric Medicine, Baqai Medical University, Karachi, PAK; 5 Pediatric Medicine, Pakistan Navy Station (PNS) Shifa, Karachi, PAK; 6 Pediatric Medicine, Jagadguru Sri Shivarathreeshwara Medical College, Mysore, IND; 7 Community Medicine, Allama Iqbal Medical College, Lahore, PAK; 8 Internal Medicine, People’s University of Medical and Health Sciences for Women, Nawabshah, PAK; 9 Internal Medicine, Adam University, Bishkek, KGZ; 10 Medicine, GSM Medical Centre, Dubai, ARE

**Keywords:** asthma control, environmental exposure, indoor pollution, pediatric asthma, recurrent wheezing

## Abstract

Background: Asthma is among the most prevalent chronic respiratory conditions in childhood, and environmental causes are suspected mainly to play a role in poor asthma control. Despite the worldwide evidence, localized investigations appraising the ecological risk factors and consequences of asthma among hospitalized children having recurrent episodes of wheezing are unavailable in Pakistan. The current study investigates the relationship between environmental exposures and asthma control in this high-risk pediatric population.

Methods: This cross-sectional study was conducted between February 2025 and May 2025 in various hospitals across Pakistan. Convenience sampling was used to enroll 385 hospitalized children aged 5-17 years with a history of recurrent wheezing. Three instruments, namely a demographic form, the Asthma Control Questionnaire (ACQ), and the International Study of Asthma and Allergies in Childhood (ISAAC) Environmental Questionnaire, were used to collect data through structured caregiver interviews. IBM SPSS version 26 (Armonk, NY: IBM Corp.) was used in statistical analyses, which included descriptive statistics, t-tests, ANOVA, Pearson correlation, and multiple linear regression, to assess the correlations between environmental exposures and asthma control.

Results: The sample (n=385) included male (206, 53%) and female (179, 47%) participants. Multiple linear regression analysis revealed that poorer asthma control was significantly predicted by higher environmental risk scores (β = -0.120, p=0.006), younger age (β=0.115, p=0.003), and female gender (β=0.110, p=0.009), indicating that females had worse asthma control than males. Other significant predictors included indoor smoking (β=0.140, p=0.003), use of wood/coal fuel (β = -0.170, p=0.003), and pet exposure (β=0.130, p=0.011). A weak but statistically significant positive correlation was found between environmental risk and asthma control scores (r=0.131, p=0.010), suggesting that greater environmental exposure was associated with poorer asthma control.

Conclusion: This study found statistically significant but modest associations between environmental exposures, such as indoor smoking, pet presence, and biomass fuel use, and asthma control in hospitalized children with recurrent wheezing. The findings underscore the necessity for targeted public health interventions and culturally sensitive caregiver education programs to mitigate modifiable environmental risk factors in pediatric asthma management in Pakistan.

## Introduction

Asthma is a complicated Th2-cytokine-mediated inflammatory disorder that causes airway hyperresponsiveness, remodeling, and chronic inflammation. There is an immune cell and cytokine-infection interaction that plays a role in structural lung changes, and recent developments allow more specific treatments [1,2]. Asthma is the leading chronic respiratory disease of childhood, with an estimated prevalence of approximately 14% worldwide, and still less than optimal outcomes with avoidable mortalities annually. Issues related to diagnosis lead to both over- and underdiagnosis, making proper management difficult [3].

Severe childhood asthma that is usually unresponsive to high-dose therapies is a heterogeneous disease with diverse clinical and inflammatory phenotypes. Better results will be achieved when there is proper diagnosis, modifiable factors are recognized, and treatment is administered based on the phenotype [4]. The global prevalence of asthma is estimated to be 334 million, including a high prevalence in children and a significant variation among countries. The prevalence of symptoms is high in high-income countries, but severe asthma symptoms appear more often in low- and middle-income countries, where the total burden is also increasing [5].

Environmental exposures such as secondary smoke, mould, air pollution, inhaled allergens, dietary deficiencies, and unflued heaters are all consistently associated with risks of asthma, symptomatology, and exacerbations in young children. Reduction of these exposures is crucial for enhancing asthma control and minimizing long-term health risks [6,7]. The prevalence and morbidity of asthma are higher in urban children, mainly due to greater exposure to indoor allergens (such as cockroaches and mice) and pollutants, including particulate matter (PM) and nitrogen dioxide (NO₂). The severity of asthma is also worsened by modifiable risk factors such as poor nutrition, deficiency of vitamin D and folate, and obesity, which can be intervention points [8].

The probability of children having a family history of asthma was highly associated with exposure to secondhand tobacco smoke (SHS) and keeping pets in the house. Such results indicate that environmental triggers and lifestyle experiences could contribute to the development of asthma in genetically susceptible children [9]. In children, asthma is strongly associated with respiratory symptoms and allergic diseases, including hay fever, atopic dermatitis, and reactions to dust and pollen. There were also a higher number of environmental exposures, such as mold and dampness, as well as tobacco smoke, that were prominent in children who had asthma [10].

Wheezing in early childhood is a frequent recurrent problem and could be a predictor of asthma. Nonetheless, it remains a clinical challenge to predict precisely which children will develop asthma [11]. Children who have a history of parental asthma or allergies and have frequent wheeze in early life have a much higher risk of having asthma by the age of seven years. Wheeze on multiple occasions before the age of three years was associated with a 12-fold increased risk, indicating the need for close follow-up in the high-risk group of children [12]. Non-invasive markers, including serum eosinophil-derived neurotoxin (EDN), eosinophil count, and exhaled nitric oxide, hold promise in evaluating both recurrent wheezing and asthma in preschool children. Nevertheless, clinical reliability requires standardized sample-collection methods and additional validation [13].

Recurrently wheezing children hospitalized are a vulnerable group, whose asthma may be more severe or less controlled. Characterization of environmental risk factors related to poor asthma control in this group is crucial for the next stage of designing targeted interventions to achieve improved morbidity and healthcare utilization. Given the widespread use of biomass fuels, indoor smoking, and other household exposures in Pakistan, this study was designed to assess how these environmental factors are associated with asthma control in hospitalized children.

This study examined the relationship between environmental risk factors and asthma control in hospitalized children with recurrent wheezing episodes, with the primary aim of informing clinical practice and population health strategies by identifying factors contributing to poor asthma outcomes and promoting improved pediatric asthma management. It specifically focused on determining the relationship between environmental exposures and the degree of asthma control in hospitalized children with recurrent wheezing in a Pakistani medical facility. The secondary aim was to identify prevalent environmental exposures - including indoor smoke, allergens, air pollution, and housing characteristics - and to evaluate their association with asthma control and hospitalization rates. Additionally, the study explored parental knowledge of asthma triggers and household practices, considering the cultural and socioeconomic context of the local population.

Rationale

Although several studies of international significance have examined how environmental factors influence the control of asthma in children, limited context-based information is available in Pakistan. Practices and beliefs, socioeconomic status, housing quality, and environmental exposures in Pakistan differ significantly from those in high-income countries. For example, the habitual use of biomass fuels for cooking, crowded living conditions, inadequate ventilation, and exposure to outdoor pollution, such as traffic and industrial pollution, may have a distinct impact on the outcome of asthma in Pakistani children. In addition, indoor smoking, burning incense, and harboring pets indoors without understanding the control of allergens are other practices that make the control of asthma much harder in this group of individuals.

Such environmental and cultural factors may have a significant impact on the disease control of hospitalized children with recurrent wheezing, who are a quite vulnerable group. Nevertheless, a typical clinical evaluation in Pakistan is not accompanied by an assessment of the environment and patient education based on local risk factors. This study was conducted in a Pakistani context to identify the most appropriate and modifiable environmental factors associated with poor asthma control, enabling the development of culturally sensitive and locally viable strategies to address pediatric respiratory health and the burden of asthma hospitalizations.

## Materials and methods

Study design and methods

A cross-sectional design study was employed to investigate the relationship between environmental risk factors and asthma control in children who experience recurrent wheezing. The sample was selected from various hospitals in Islamabad, Pakistan, including both governmental and non-governmental medical institutions, to ensure the representation of individuals from diverse socioeconomic and cultural backgrounds. Data were collected from Kulsom Hospital, Shifa International Hospital, and Pakistan Institute of Medical Sciences (PIMS) Hospital. The information was gathered from the children while they were in the hospital, based on a structured questionnaire that focused on environmental exposures and the level of asthma control.

Trained research staff approached parents or primary caregivers during regular ward rounds or visiting hours. The study's purpose was clearly described in the local language, and caregivers were given time to ask questions and then decide whether to participate. Only volunteers who gave written or verbal informed consent were recruited for the study. The caregivers were either interviewed by the research team or completed the questionnaire, depending on their preference and literacy level. Respectful interaction with families and high-quality data corresponding to real-life situations in Pakistani households were guaranteed by this approach.

Sample size and technique

The exact number of children admitted to hospitals with recurrent wheezing in Pakistan is unknown; thus, the population was assumed to be infinite in this study. The sample was determined through the below formula.

$n = \frac{Z^2 \cdot p (1 - p)}{d^2}$

Where Z is the standard value by the required level of confidence, p is the estimated proportion by past research, and d is the acceptable margin of error in this equation. The z-value used was 1.96, which corresponds to a 95% level of confidence, and the margin of error (d) was set at 0.05. The researchers chose p=0.50 to obtain the maximum sample size based on the results of previous regional studies that showed approximately half of children with asthma had poor control [14].

A convenience sampling method was used to recruit participants from pediatric inpatient wards of tertiary care hospitals in Islamabad, Pakistan. Caregivers of eligible children were approached by trained research assistants during ward rounds or visiting hours and informed about the purpose of the study. A total of 410 caregivers were approached. The participants were recruited from three hospitals: Shifa International Hospital (150 approached, 140 included), PIMS Hospital (160 approached, 150 included), and Kulsom Hospital (100 approached, 95 included). Out of this number, 385 individuals agreed to fill out the questionnaire, resulting in a response rate of approximately 94%. The non-response rate was approximately 6% (25/410), mainly due to caregiver refusal or incomplete clinical records. Children with incomplete clinical data or whose caregivers declined participation (n=25) were excluded from the final analysis. This approach ensured that only children with confirmed recurrent wheezing and complete exposure and outcome data were included.

Inclusion criteria

The study included children between five and 18 years of age who had been hospitalized with a clinical history of recurrent wheezing. Eligible participants had to be hospitalized due to asthma-related symptoms during the data collection period and must have had a caregiver available who could provide informed consent on their behalf. The caregiver was also required to be in a position to answer and comprehend questions, either in Urdu or English, since data were to be gathered using structured interviews or questions.

Exclusion criteria

Children with other chronic respiratory diseases, such as cystic fibrosis or bronchopulmonary dysplasia, were excluded from the study. Also excluded were those with congenital anomalies of the respiratory system, severe developmental delays, or neurological conditions that may have confounded breathing patterns or communication. Moreover, cases were disqualified when caregivers refused participation, were inaccessible throughout the hospital stay, or when vital clinical information was absent or unclear.

Data collection tools

To gather appropriate data for this study, a structured questionnaire was developed consisting of three main parts: demographic data, asthma control, and environmental exposure. The questionnaire aimed to provide a comprehensive understanding of the factors that influence the control of asthma in children admitted to hospitals with recurrent wheezing.

Demographic information

The initial part of the questionnaire contained demographic and clinical background data to determine if age, gender, parental education, and other factors were associated with asthma control. Additional clinical and contextual variables were also included to facilitate a more comprehensive analysis. These were the age of the child at the time of the first asthma diagnosis, past medical hospitalization due to asthma or wheezing, the presence of other chronic respiratory illnesses, and every known allergy. Environmental features of the households were also noted, including the type of house, the number of individuals per room, whether pets were kept in the house, and the primary fuel used for cooking. These variables have been chosen because they have some possible significance to the outcome of asthma and environmental exposure, as suggested by past research.

Asthma control questionnaire (ACQ)

The second section involved administering the Asthma Control Questionnaire (ACQ), a validated tool developed by Dr. Elizabeth Juniper in 1999 to assess asthma control, particularly in clinical and research settings. The ACQ measures symptom experience in the past week using six symptom-based questions and one question about the use of short-acting bronchodilators. All responses are presented on a seven-point Likert rating scale, with lower scores indicating better asthma control. When spirometry is involved, a lung functioning item can also be included. The total ACQ score is calculated by averaging all item scores, with a higher score indicating poorer control of asthma. The scores can be interpreted as follows: a score of 0.75 or less implies well-controlled asthma, a score of 1.5 or more describes poorly controlled asthma, and a score between 1.5 and 0.75 represents partially controlled asthma. The tool is well-validated in adults and pediatrics. Its internal consistency has already been established in various studies, with a Cronbach's alpha value of 0.76-0.89, which is classified as good to excellent reliability [15]. The permission to use the ACQ in this study was received officially.

ISAAC environmental questionnaire

The third section was based on the Environmental Questionnaire of the International Study of Asthma and Allergies in Childhood (ISAAC), which was developed in the early 1990s by the ISAAC Steering Committee to facilitate a standardized approach to evaluating environmental risk factors for asthma and allergies across diverse populations. The questionnaire contained questions about household exposures, including indoor smoking, ownership of pets, cooking fuel used, dampness or mould in the dwelling, and outdoor pollution, which are among the common factors associated with asthma symptoms. Items were presented in categorical or Likert-type formats, and each response option was assigned a numerical score based on its associated level of risk for asthma. Responses were typically scored on a scale of 1 (low or no exposure) to 3 (high exposure), though some items had binary scoring (e.g., yes=1, no=0), depending on the question format. Specific examples of scoring included: "Does anyone smoke inside the house?" (yes=1, no=0); "Is there visible mould or dampness in the dwelling?" (never=1, sometimes=2, often=3); "What type of cooking fuel is mainly used in your house?" (electricity or gas=1, biomass fuels like wood or coal=3); "Are there pets living inside the house?" (no=0, yes=1); and "How often does the child consume fast food?" (less than once a week=1, 1-2 times per week=2, 3 or more times per week=3). Total environmental risk scores were calculated by summing individual item scores, where a higher total indicated greater cumulative ecological risk. It was previously utilized extensively in global research, even in low- and middle-income countries, and demonstrated acceptable reliability in children. Cronbach's alpha values reported for domains of environmental exposure generally ranged from 0.70 to 0.82 [16]. Permission has been granted to use the ISAAC Environmental Questionnaire. An overview of the asthma assessment tools, including their reliability and key features, is provided in Table 1.

**Table 1:** Psychometric properties and key characteristics of asthma assessment instruments.

Instrument	Asthma Control Questionnaire (ACQ)	ISAAC Environmental Questionnaire
Developer	Dr. Elizabeth Juniper [15]	ISAAC Steering Committee [16]
Year developed	1999	The early 1990s
Purpose	Assessment of the level of asthma control in clinical practice and research	To measure environmental risk factors relating to asthma and allergies
Target population	Adults and pediatric populations	Primarily children
Structure	6 Symptoms questions + 1 question about use of bronchodilators (7-point Likert scale); optional lung function question;	Environmental exposure questions (e.g., indoor smoking, pets, fuel, mold, pollution)
Reliability (Cronbach’s alpha)	0.76-0.89 (good to excellent reliability)	0.70-0.82 (acceptable reliability across domains)
Validation status	Validated in both adult and pediatric populations	Widely used and validated globally, including in low- and middle-income countries

Procedure

The participants were recruited from the pediatric wards of tertiary care hospitals, with informed consent obtained from their parents or legal guardians. The data collection began in February 2025 and continued until May 2025, spanning a period of four months. The caregivers participated in the study through either face-to-face interviews with trained data collectors or a self-completion questionnaire, depending on their preference and literacy level. The dataset was stripped of all identifying information to ensure confidentiality and comply with ethical standards. Using this method made it possible to gather trustworthy and respectful data involving families with different socioeconomic and cultural backgrounds.

Statistical analysis

IBM SPSS Statistics version 26 (Armonk, NY: IBM Corp.) was used to analyze the data. Descriptive statistics, including means, standard deviations, frequencies, and percentages, were applied to characterize the participants. The Kolmogorov-Smirnov and Shapiro-Wilk tests were conducted to assess the normality of the data distribution. The correlation between the Environmental Questionnaire and the Asthma Control Questionnaire was determined by Pearson correlation analysis. An independent t-test was performed to determine whether there were any differences in scores between the Environmental Questionnaire and Asthma Control Questionnaire for males and females, as well as for those with and without hospitalization due to asthma/wheezing. One-way ANOVA was used to explore the differences in mean scores of the Environmental Questionnaire and Asthma Control Questionnaire between groups classified by parental education level and primary cooking fuel at home.

Additionally, a multiple linear regression analysis was conducted to examine the predictive relationship between the Environmental Questionnaire scores and the Asthma Control Questionnaire scores. Lastly, the relationship between the type of primary caregiver and the educational level of parents, as well as between the primary caregiver and the predominant cooking fuel at home, was examined using chi-square tests. All statistical tests were applied, with a significance level of p<0.05 used to determine factors affecting asthma control and environmental health outcomes.

Ethical considerations

The research was conducted according to the established ethical guidelines for research involving human subjects. The study protocol was accepted by the Institutional Review Board of Mindwave Research Center, Islamabad (#IRB-2025-0016), which is why the main ethical principles, including respect for persons, beneficence, and confidentiality, were considered during the study. Before participation, caregivers were fully informed about the study's purpose, procedures, risks involved, and benefits. All parents or legal guardians signed a written informed consent before the collection of any data. The participation procedure was entirely voluntary, and respondents could leave the study at any point without repercussions.

Confidentiality was assured by deleting all personal identifiers and storing the data securely. Questionnaires with incomplete or missing responses were reviewed as needed. In cases of non-response to crucial sections, especially those related to the Asthma Control Questionnaire, the forms were excluded from the final analysis to preserve the quality and integrity of the data.

## Results

Table 2 demonstrates the demographic features of the participants (n=385). Most of the participants fell within the age group of 8-10 years (177, 46%), followed by 5-7 years (150, 39%), and a low number of participants were in the age group of 11-13 years (47, 12%) and 14-17 years (11, 3%). There were 206 males (53%) and 179 females (47%). Among primary caregivers, the majority of children were living with their fathers (176, 47%), mothers (107, 28%), and grandparents (102, 26%). In terms of parental education, 142 (37%) of parents finished primary school, 110 (29%) finished secondary school, and 91 (24%) parents had no formal education. Most of the children had been hospitalized with asthma or wheezing (220, 57%), and the large majority had other chronic respiratory diseases (268, 70%). The most frequent age at first asthma diagnosis was 2-5 years (169, 44%), and 255 (66%) of children had known allergies. Smoking was done indoors in 257 (67%) of households, and the primary cooking fuels were electricity (159, 41%) and gas (153, 40%). Lastly, 226 (59%) children were in homes with pets. These attributes give an in-depth insight into the determinants of respiratory health among the respondents.

**Table 2:** Demographic characteristic of participant (n=385). f: frequency

Variables	f	%
Age		
5-7 years	150	39
8-10 years	177	46
11-13 years	47	12
14-17 years	11	3
Gender		
Male	206	53
Female	179	47
Primary caregiver		
Mother	107	28
Father	176	47
Grandparents	102	26
Parental educational level		
No formal education	91	24
Primary school	142	37
Secondary school	110	29
Has the child been hospitalized for asthma or wheezing before?		
Yes	220	57
No	165	43
Does the child have any other chronic respiratory disease?	-	-
Yes	268	70
No	117	30
Age at first asthma diagnosis		
<2 years	104	27
2-5 years	169	44
6-10 years	112	29
Does the child have any known allergies?		
Yes	255	66
No	130	34
Does anyone smoke inside the home?		
Yes	257	67
No	128	33
Primary cooking fuel used at home		
Gas	153	40
Electricity	159	41
Wood/coal	62	16
Other	11	3
Are there pets living in the home?		
Yes	226	59
No	159	41

Table 3 describes the outcome of the testing of normality and the results of the assessment of normality of filling in the Environmental and Asthma Control Questionnaires on both the Kolmogorov-Smirnov and Shapiro-Wilk tests. In the case of the Environmental Questionnaire, the Kolmogorov-Smirnov test reported a statistic of 0.032 (p=0.200), and the Shapiro-Wilk test returned a statistic of 0.992 (p=0.076). Likewise, the Asthma control questionnaire exhibits a Kolmogorov-Smirnov statistic of 0.027 (p=0.200) and a Shapiro-Wilk statistic of 0.995 (p=0.182) as well. The p-values were greater than 0.05 in both instances, so both distributions of the two variables were not significantly different from normal. Hence, we can assume that both questionnaire data sets are normally distributed and the parametric statistics analysis is reasonable.

**Table 3:** Tests of normality for Environmental and Asthma Control Questionnaires. df: degree of freedom Normality was assessed using the Kolmogorov-Smirnov and Shapiro-Wilk tests. A p-value of >0.05 indicated normal distribution (suitable for parametric tests), while a p-value ≤0.05 indicated non-normal distribution (requiring non-parametric tests).

Variables	Kolmogorov-Smirnov test			Shapiro-Wilk test		
	Statistic	df	p-Value	Statistic	df	p-Value
Environmental Questionnaire	0.032	385	0.200	0.992	385	0.076
Asthma Control Questionnaire	0.027	385	0.200	0.995	385	0.182

Table 4 demonstrates the intercorrelation between the Environmental Questionnaire and the Asthma Control Questionnaire. These two variables were weakly but significantly correlated (r=0.131, p=0.010), indicating a weak, positive, and significant correlation. It implies that the higher the scores on the Environmental Questionnaire, the higher the scores on the Asthma Control Questionnaire rise slightly. This significant value shows that there is some correlation between the two variables; however, the low correlation value means that there are other variables that could also contribute to the results of asthma control.

**Table 4:** Intercorrelation between study variables. *P<0.001 considered significant. A Pearson correlation was used to assess the relationship between variables.

Variable	Environmental Questionnaire	Asthma Control Questionnaire	p-Values
Environmental Questionnaire	-	0.131	0.010^*^
Asthma Control Questionnaire	0.131	-	0.010^*^

Table 5 shows a comparison between males and females in two questionnaires: the Environmental Questionnaire and the Asthma Control Questionnaire. Regarding the summary of the Environmental Questionnaire, the mean±standard deviation for males was 51.57±3.31 and for females 51.08±3.36, with a t-value of 1.422 and a p-value of 0.078, indicating an insignificant gender difference (p>0.05). In the same manner, the results obtained in the Asthma Control Questionnaire showed a mean score of 21.57±4.06 in the male respondents and 22.41±4.56 in the female respondents, with a t-value of 1.924 and a p-value of 0.056, thus showing a tendency that borders on p<0.05 without reaching the critical value. The effect sizes of both comparisons were also small, with coefficients of 0.15 and 0.20, which may indicate insignificant gender differences in these measures.

**Table 5:** Comparison among variables (gender). LL: lower limit; UL: upper limit; Cl: confidence interval; t: independent t-test

Variables	Male (n=206), mean±SD	Female (n=179), mean±SD	t-Value	p-Value	Cl 95% LL	UL	Cohen’s D
Environmental Questionnaire	51.57±3.31	51.08±3.36	1.422	0.078	-0.0215	0.741	0.15
Asthma Control Questionnaire	21.57±4.06	22.41±4.56	1.924	0.056	-1.298	0.398	0.20

Table 6 presents a comparison between participants who experienced asthma or wheezing hospitalization and those who did not, using two questionnaires: the Environmental Questionnaire and the Asthma Control Questionnaire. In the case of the Environmental Questionnaire, both the mean score of the yes (51.10±3.17) group and the no (51.66±3.54) group were reasonably close to each other, with a t-value of 1.631, p-value of 0.104, which serves to indicate that no significant difference occurred between the two groups (p>0.05). The small effect size of -0.17 suggests that Cohen's d is also small, indicating that the differences are not substantial. In the case of the Asthma Control Questionnaire, the difference between the yes group and the no group was 21.88±4.35 and 22.07±4.28, respectively; the t-value stood at 0.427, and the p-value of 0.678 showed no significant difference (p>0.05). The value of Cohen's d, -0.04, further indicates an insignificant effect size between groups. Overall, these results suggest that there are no substantial concerns regarding differences between groups in either questionnaire.

**Table 6:** Comparison among variables (hospitalized due to asthma or wheezing). LL: lower limit; UL: upper limit; Cl: confidence interval; t: independent t-test

Variables	Yes (n=220), mean±SD	No (n=165), mean±SD	t-Value	p-Value	Cl 95% LL	UL	Cohen’s D
Environmental Questionnaire	51.10±3.17	51.66±3.54	1.631	0.104	-1.230	0.118	-0.17
Asthma Control Questionnaire	21.88±4.35	22.07±4.28	0.427	0.678	-1.062	0.680	-0.04

Table 7 compares the mean scores of two questionnaires (Environmental Questionnaire and Asthma Control Questionnaire) across four groups of parental education levels - no formal education, primary school, secondary school, and higher education. For the Environmental Questionnaire, the mean scores range from 50.64±3.07 in the "no formal education" group to 51.73±3.63 in the "secondary school" group, with a p-value of 0.119 and an F-ratio of 1.965, indicating no significant differences between the groups and a tiny effect size (η²=0.015). Similarly, for the Asthma Control Questionnaire, mean scores range from 21.69±4.19 in the "no formal education" group to 22.13±4.27 in the "secondary school" group, with a p-value of 0.911 and an F-ratio of 0.175, further confirming the absence of significant differences. Both questionnaires demonstrate minimal variance based on parental education level, suggesting that parental education does not have a substantial impact on the outcomes measured in this study.

**Table 7:** Comparison of variables (parental educational level). F: ratio of variance between groups to within groups; η^2^: effect size One-way ANOVA was used to assess differences in mean scores across multiple categories, such as parental education and type of cooking fuel used.

Variables	No formal education (n=91), mean±SD	Primary school (n=142), mean±SD	Secondary school (n=110), mean±SD	Higher education (n=42), mean±SD	p-Value	F (3,381)	η^2^
Environmental Questionnaire	50.64±3.07	51.42±3.34	51.73±3.63	51.60±2.93	0.119	1.965	0.015
Asthma Control Questionnaire	21.69±4.19	21.99±4.45	22.13±4.27	22.00±4.36	0.911	0.175	0.001

Table 8 compares the mean scores of the Environmental Questionnaire and the Asthma Control Questionnaire across four groups based on the primary cooking fuel used at home: gas, electricity, wood/coal, and others. For the Environmental Questionnaire, the mean scores range from 50.45±2.88 in the "other" fuel group to 51.77±3.62 in the "wood/coal" group, with a p-value of 0.598 and an F-ratio of 0.626, indicating no significant differences between the groups and a small effect size (η²=0.034). Similarly, for the Asthma Control Questionnaire, mean scores range from 21.55±4.25 in the "electricity" group to 22.55±4.25 in the "other" fuel group, with a p-value of 0.462 and an F-ratio of 0.860, further confirming the lack of significant differences. Both questionnaires show minimal variance based on the primary cooking fuel used at home, suggesting that the type of fuel does not have a substantial impact on the outcomes measured in this study.

**Table 8:** Comparison of variables (main cooking fuel used at home). F: ratio of variance between groups to within groups; η^2^: effect size One-way ANOVA was used to assess differences in mean scores across multiple categories, such as parental education and type of cooking fuel used.

Variables	Gas (n=153), mean±SD	Electricity (n=159), mean±SD	Wood/coal (n=62), mean±SD	Other (n=11), mean±SD	p-Value	F (3,381)	η^2^
Environmental Questionnaire	51.30±3.39	51.28±3.21	51.77±3.62	50.45±2.88	0.598	0.626	0.034
Asthma Control Questionnaire	22.29±4.47	21.55±4.25	22.08±4.11	22.55±4.25	0.462	0.860	0.051

Table 9 presents the multiple linear regression results for the variables of the Asthma Control Questionnaire scores (n=385). The analysis indicates that certain factors play a crucial role in the process of asthma control, such as Environmental Questionnaire scores (β = -0.120, p=0.006), where higher environmental scores imply somewhat reduced asthma control. There is a positive relationship between age (β=0.115, p=0.003) and gender (β=0.110, p=0.009) and improvement in asthma control; being older and male leads to minimal gains. Asthma control is negatively correlated with smoking indoors (β=0.140, p=0.003) and even with the use of certain cooking fuels (β = -0.170, p=0.003), particularly wood and coal. Moreover, the presence of pets in the house (p=0.011, 0.130) is also associated with better asthma control. All the predictors are statistically significant, and smoking/cooking fuels have the most negative impact, whereas age, gender, and pets appear to have an overall positive effect on asthma control.

**Table 9:** Multiple linear regression analysis predicting Asthma Control Questionnaire scores (n=385). *P<0.01 considered significant. B: coefficient; SE: standard error; β: standardized coefficient; LL: lower limit; UL: upper limit

Variables	B	95% Cl LL	UL	SE	β	p-Value
Constant	29.10	22.90	35.30	3.15	-	<0.001^*^
Environmental Questionnaire	-0.165	-0.282	-0.048	0.060	-0.120	0.006^*^
Age	0.630	0.216	1.044	0.210	0.115	0.003^*^
Gender	0.950	0.243	1.657	0.360	0.110	0.009^*^
Smoking inside the home	1.180	0.412	1.948	0.390	0.140	0.003^*^
Main cooking fuel	-0.910	-1.499	-0.321	0.300	-0.170	0.003^*^
Pets in the home	1.020	0.236	1.804	0.400	0.130	0.011^*^

Table 10 shows the descriptive analysis of the significant demographic characteristics, like the primary caregiver, level of education attained by parents, and the primary cooking fuel at home. Of the entire sample (n=385), the most frequent primary caregivers were fathers (n=176), then mothers (n=107), and finally grandparents (n=102). There was also a substantial correlation between parental education level and primary caregiver (p<0.001, chi-square=6.11). Out of the mothers, there were 27 without any formal education, 43 with a primary education, 27 with secondary school education, and 10 with a higher education. Of fathers, 47 were not educated, 63 had primary education, 48 had secondary education, and 18 had higher education. Of the grandparents, 17 were educated, 36 were educated at the primary level, 35 at the secondary level, and 14 at the higher level. There was a statistically significant association between primary caregiver and main cooking fuel used at home (χ²=12.9, p=0.04). Gas was utilized in 65 households, electricity in 84, and wood/coal in 26, when the fathers were the primary caregivers. In households that had mothers as heads, gas was utilized in 46, electricity in 36, and wood/coal in 18. In grandparent-headed households, 42 used gas, 39 used electricity, and 18 used wood and coal. These results highlight the fact that the identity of the primary caregiver significantly influences educational level and domestic environmental exposures.

**Table 10:** Descriptive statistics of demographic variables (primary caregiver, parental educational level, main cooking fuel used at home). *P<0.01 considered significant. **P<0.05 considered significant. f: frequency P-values were calculated using the chi-square test.

Variables	f	No formal education	Parental educational level primary	Secondary	Higher education	p-Value	χ²	Gas	Main cooking fuel is electricity	Wood/coal	Other	p-Value	χ²
Primary caregiver	-	-	-	-	-	<0.001^*^	6.11	-	-	-	-	0.04^**^	12.9
Mother	107	27	43	27	10	-	-	46	36	18	-	-	-
Father	176	47	63	48	18	-	-	65	84	26	-	-	-
Grandparents	102	17	36	35	14	-	-	42	39	18	-	-	-

## Discussion

This study aimed to investigate the relationship between environmental risk factors and asthma control in children hospitalized with recurrent wheezing in Pakistan. In our study, we identified a small but significant association between environmental exposures and asthma control, suggesting that environmental factors may influence asthma control. This aligns with prior research, which highlights that both indoor and outdoor modifiable ecological factors are linked to asthma symptoms and should be targeted in customized care plans [17].

This study found that males had slightly higher environmental exposure scores than females. However, this difference was not statistically significant. This may reflect gender-based differences in household roles, exposure patterns, or cultural practices, as also suggested by previous studies [18]. Additionally, women were identified as having worse asthma control than men. This is consistent with other studies, which have demonstrated that asthma is more severe and less controlled in girls and women, which may reflect hormonal effects and gender differences in environmental exposures [19].

There was no statistically significant difference in environmental exposure between hospitalized and non-hospitalized patients in our study. This suggests that hospitalization due to asthma or wheezing did not have a substantial impact on environmental exposure, which contrasts with previous studies that have linked higher exposure to asthma triggers with increased hospitalization and worse outcomes [20]. Our study found no significant difference in asthma control between hospitalized and non-hospitalized patients, which contrasts with a previous study that showed poor asthma control is strongly associated with increased hospitalizations and healthcare utilization. This discrepancy may be due to differences in asthma control measurement or other factors not accounted for in this study [21].

In this study, no significant differences in environmental awareness were observed across different parental education levels, suggesting that factors other than education may influence ecological behaviors. This contrasts with other studies, which demonstrate that educational interventions can lead to meaningful changes in behavior, highlighting that education alone may not always drive significant shifts in environmental awareness or asthma control [22]. In our research, no significant correlation was found between the education level of parents and asthma control, as measured by the Asthma Control Questionnaire (p=0.911). This finding is contrary to earlier studies, which reported that higher parental education is associated with improved asthma control among children [23]. The difference could be attributed to differences in study design/population characteristics or other unmeasured factors related to asthma management besides parental education.

No statistically significant difference was noted in our study regarding environmental exposure based on the type of cooking fuel used. However, other reports suggest that cleaner cooking fuels, such as liquefied petroleum gas (LPG), can yield improved environmental outcomes, primarily through enhanced kitchen ventilation and the adoption of new stoves [24]. Our study did not reveal any significant correlation between the kind of cooking fuel used at home and asthma outcomes. On the contrary, earlier studies have indicated that exposure to open fires during cooking is associated with an increased likelihood of asthma symptoms among children, especially in areas where these fuels are widely used [25].

Participants with poorer asthma control tended to report higher environmental awareness scores. This suggests that individuals with more severe symptoms may develop a heightened awareness of environmental triggers. This is consistent with past literature that emphasizes the importance of the physical and social environment in asthma morbidity and highlights the relevance of modifying ecological exposures as a strategy for enhancing asthma control [17]. However, reverse causality is possible and should be explored further in longitudinal research.

Additionally, this study also indicates that gender is a significant factor in the management of asthma, and males have better control of asthma than females. This is in line with the current literature showing that females are more affected by asthma, with an increasing prevalence suggesting the need to consider gender when managing asthma [19]. Furthermore, age had a positive impact on asthma control in this study, with better asthma management observed in older individuals. This finding aligns with a study that reported a similar pattern, where asthma control was more effective among older children than younger children, suggesting a possible role of age in asthma management [26]. We found that pet exposure had no significant impact on asthma control and symptoms. Nevertheless, one study suggested that early exposure to pets resulted in an increased prevalence of asthma, especially among children with parental atopy. This discrepancy can have various causes, including differences in environmental influences, genetic factors, or variations in healthcare practices in other regions [27].

Taken together, these findings suggest a multifactorial approach to controlling asthma in children and highlight the need to address environmental and socioeconomic factors during clinical evaluation and population health strategies. However, given the small effect sizes and observational design, the findings should be interpreted with caution. It is essential to note that our regression analysis did not account for key confounding factors, such as medication adherence, socioeconomic status, and nutritional status, which may also impact asthma control outcomes.

Limitations

The current research is associated with several limitations that must be considered when explaining the results. First, the cross-sectional nature of the study limits the possibility of making causal inferences between environmental exposures and asthma control; longitudinal studies would be required to define temporal relations. Second, a significant portion of the data relied on self-reported answers from caregivers, which could lead to recall bias or social desirability bias, particularly on sensitive topics such as indoor smoking or pet ownership. Furthermore, convenience sampling based on hospital-based sampling from urban settings may limit the generalizability of the findings to the broader pediatric population, particularly rural children, who may have different exposure profiles and access to care. The absence of objective clinical parameters, such as spirometry or inflammatory markers, also represents another limitation, as it would have reinforced the evaluation of asthma control. Although significant predictors were identified using multiple linear regression, there are still essential variables that were not quantified in this study, such as socioeconomic status, medication adherence, nutritional status, and access to care. Lack of these data can hinder the capacity to fully understand the role of environmental exposures in asthma control, as these factors could be potential confounders.

Future directions

Future studies should focus on overcoming the limitations listed above and building upon existing findings. Longitudinal studies following children help establish causal relationships between environmental risk factors and the development or control of asthma. Adding objective clinical measures, such as lung function tests and biomarker analysis, would enhance the accuracy and reliability of assessing asthma control. Wider sampling to cover rural and underserved communities would improve the applicability of the findings to a broader range of populations with diverse socioeconomic and geographic backgrounds. Moreover, intervention-oriented research on modifiable risk factors, including reducing household smoke exposure, improving indoor air quality, and enhancing caregiver education, may also be translated into practical measures to ensure better outcomes for pediatric asthma. Additional investigations of psychosocial issues, such as caregiver stress and knowledge of asthma triggers, could also provide important insights regarding holistic management of asthma within culturally and resource-diverse environments. Future research should incorporate socioeconomic status, medication adherence, and other potential confounding factors to better isolate the impact of environmental exposures on asthma control.

## Conclusions

This study highlights a statistically significant but modest association between environmental risk factors and asthma control among children with recurrent wheezing admitted to hospitals in Pakistan. Notably, indoor smoking, pet exposure, and the use of biomass fuels for cooking were significant predictors of poorer asthma control. Additionally, female gender and younger age were associated with worse asthma outcomes. While these findings support the role of modifiable household factors, the cross-sectional design limits the ability to establish causal relationships. Therefore, the associations should be handled with caution. The results indicate the need to focus on modifying household and lifestyle factors that can be achieved by implementing locally specific public health programs as soon as possible. Specifically, culturally sensitive caregiver education programs, smoke-free and asthma-friendly policies, and improved housing may make a significant contribution to the improved management of asthma in vulnerable pediatric groups. It's essential to emphasize the importance of a multifaceted approach that combines environmental, clinical, and social interventions. This comprehensive strategy reassures us that we are addressing all aspects of the problem. Conclusively, the burden of asthma should be reduced and the quality of life of affected children improved through this multifaceted approach.
